# Incidence and Survival Rates for Female Malignant Germ Cell Tumors: An Institutional Review

**DOI:** 10.7759/cureus.24497

**Published:** 2022-04-26

**Authors:** Afshan Saeed Usmani, Iqra Yasin, Rehan B Asif, Nazish Kahlid, Aamir Syed

**Affiliations:** 1 Department of Surgical Oncology, Shaukat Khanum Memorial Cancer Hospital and Research Centre, Lahore, PAK; 2 Department of Surgical Oncology: Gynecologic Oncology, Shaukat Khanum Memorial Cancer Hospital and Research Centre, Lahore, PAK

**Keywords:** uterine malignancy, survival, ovarian carcinoma, malignant germ cell tumor, chemotherapy

## Abstract

Background

Germ cell tumor survival rates have improved over the past few decades. However, there is a lack of data on survival rates and the incidence of female germ cell tumors. This study aims to determine the incidence and survival rates of female germ cell tumors in our institution.

Methodology

This retrospective cross-sectional study was carried out at Shaukat Khanum Memorial Hospital and Research Centre, and the records over 10 years, from January 2010 to December 2020 were examined. The data of 290 females with malignant germ cell tumors were selected from 1387 females with ovarian masses, and their survival records were examined. For statistical analysis, SPSS software (version 24.0; IBM Corp. Armonk, NY) was utilized. The survival analysis was determined using the Kaplan-Meier method.

Results

The mean age of patients was 21.45 ± 9.28 years. The mean duration of diagnosis was 4.53 ± 2.59 years. In 245 (84.5%) patients, ovarian malignancy was involved while uterine malignancy was observed in 44 (15.2%) cases and there was one (0.3%) case of cervical carcinoma. The most common stage at diagnosis of malignancy was IA (96 (33.1%)), followed by IIIC (58 (20.0%)), IV (56 (19.3%)) and IC (26 (9.0%)). Chemotherapy was given in 244 (84.1%) cases. Out of 290 cases, 26 (9.0%) had a recurrence of the tumor while 264 (91.0%) did not have a recurrence of the tumor. Out of 290 cases, 46 (15.9%) died during follow-up, 129 (44.4%) had disease-free survival while 115 (39.7%) were healthy till the end of the study. The mean duration of survival was 3.56 ± 2.33 years. When patients' survival was compared between treatment groups, patients who did not receive chemotherapy fared better than those who did.

Conclusion

Female germ cell tumor patients have a good overall survival rate of more than 20% after 10 years of follow-up with effective adjuvant therapy and conservative surgery. However, more research is needed to determine the long-term effects of chemotherapy on ovarian function.

## Introduction

Germ cell tumors are heterogeneous benign or malignant neoplasms that arise in the gonadal and midline extragonadal organs [1]. Ovarian germ cell tumors make up 20-25% of all ovarian neoplasms in females, however, only 3-5% of them are cancerous [2-3]. Dysgerminoma, yolk sac tumors, mixed germ cell tumors, and immature teratomas account for more than 90% of all malignant germ cell tumors. The remaining 5-10% of cell types are embryonic cancer, choriocarcinoma, and polyembryoma, which are rarely observed in pure form and have a poor prognosis [3-4].

Seminomas, which include testicular seminomas and ovarian dysgerminomas, and non-seminomas, which include histologic subtypes of yolk sac tumors, teratomas, embryonal carcinomas, and choriocarcinomas, are the two categories of germ cell tumors. Females are more likely than males to develop germ cell tumors, which are normally benign (mature teratomas or "dermoid" tumors) (0.8:1 male-to-female ratio) [5-6].

At the time of diagnosis, ovarian malignant germ cell tumors are typically big and progress quickly. Abdominal pain (87%) and an abdominal mass are the most common symptoms in adolescence (85%). Acute abdomen is seen in about 10% of patients as a result of torsion, bleeding, or tumor rupture. Abdominal distention, fever, and vaginal bleeding are less common symptoms. Symptoms are usually brief, lasting 2-4 weeks on average [7].

Ovarian malignant germ cell tumors are generally unilateral, although, in about 4.3% of patients, they might be bilateral. With pure non-dysgerminoma cell lines, bilaterality is more common in dysgerminomas and mixed germ cell tumors [8-9]. Germ cell tumor survival rates have improved considerably during the last three decades, coinciding with more aggressive surgical staging and combined modality. Germ cell tumors make for a substantially bigger proportion of ovarian neoplasms in Asia and Africa (6% of all cancers) while epithelial ovarian carcinoma is less common [10-12].

The primary treatment for most germ cell tumors is surgery. The type of surgery depends on the location and stage of the tumor. In some cases, adjuvant chemotherapy may be recommended. If the tumor has spread to other parts of the body, chemotherapy may be the primary treatment. In some cases, radiation therapy may be used. The survival of these tumors has improved considerably since the introduction of platinum-based chemotherapy in the 1980s, with five-year survival rates of 90% recorded. Gonadal cancers have also been shown to have a better prognosis than extragonadal malignancies [13]. Trends in malignant female germ cell cancers, particularly in Asian races, are poorly understood. The goal of this research was to look at trends in the incidence and survival rates of malignant ovarian germ cell tumors in the South East Asian population. This study aimed to evaluate the 10-year survival of malignant germ cell tumors in the reproductive system of females.

## Materials and methods

Study design

This was a retrospective, cross-sectional, single-institution experience.

Study setting

The study was conducted in Shaukat Khanum Memorial Hospital and Research Centre after approval from the institutional review board of Shaukat Khanum Memorial Cancer Hospital and Research Center vide letter no. Ex-17-05-21-01.

Study duration

The data for 10 years, i.e., January 2010 to December 2020, were explored.

Inclusion criteria

Females diagnosed with primary malignant germ cell tumors on histopathology were included.

Exclusion criteria

Females with non-primary malignant germ cell tumors were excluded.

Sample size

The data of 1387 females were included, as it was a time-based retrospective study.

Data collection procedure

The records of 1387 females diagnosed with ovarian masses were explored and 290 females (20.9%) females diagnosed with primary malignant germ cell tumors on histopathology were included. The data of the patients, including age, duration of diagnosis, primary tumor site, histologic subtype, treatment given, and recurrence and survival during follow-up were noted. Five and ten-years survival rates were calculated and disease-free survival was noted as survival without progression, recurrence, and death.

Statistical analysis

The data were entered and analyzed using SPSS software (version 24; IBM Corp., Armonk, IBM). Quantitative variables, i.e., age, duration of disease, and duration of survival, have been presented as mean and standard deviation. Categorical variables, i.e., gender, age groups, year-wise disease distribution, tumor grades, treatment given (chemotherapy given or not), recurrence, and survival of the patient, have been presented as frequency and percentage. Survival rate as determined by using the Kaplan-Meier method. P-value ≤ 0.05 was considered significant.

## Results

In this study, the mean age of patients was 21.45 ± 9.28 years. Out of 290 cases, eight (2.8%) were aged <5 years while the maximum numbers of patients fall in the age group 20-40 years, which is the reproductive age group. The mean duration of diagnosis was 4.53 ± 2.59 years. The maximum number of patients were enrolled during the period 2012 to 2018. In 245 (84.5%) patients, ovarian malignancy was involved while uterine malignancy was observed in 44 (15.2%) cases and there was one (0.3%) case of cervical carcinoma. The most common stage at diagnosis of malignancy was IA (96 (33.1%)), followed by IIIC (58 (20.0%)), IV (56 (19.3%)), and IC (26 (9.0%)). Chemotherapy was given in 244 (84.1%) cases while in 46 (15.9%) cases, no chemotherapy was given (Table 1).

**Table 1:** Baseline characteristics of females

	Mean ± SD, f (%)
n	290
Age (years)	21.45 ± 9.28
Age group	
<5 years	8 (2.8%)
6-10	24 (8.3%)
11-19	97 (33.4%)
20-40	147 (50.7%)
41-55	14 (4.8%)
Diagnosis of disease	
2010	1 (0.3%)
2011	20 (6.9%)
2012	24 (8.3%)
2013	25 (8.6%)
2014	36 (12.4%)
2015	42 (14.5%)
2016	36 (12.4%)
2017	37 (12.8%)
2018	33 (11.4%)
2019	9 (3.1%)
2020	27 (9.3%)
Mean duration of disease	4.53 ± 2.59
Site of tumor	
Ovary	245 (84.5%)
Uterus	44 (15.2%)
Cervix	1 (0.3%)
Grade of tumor	
I	2 (0.7%)
IA	96 (33.1%)
IB	9 (3.1%)
IC	26 (9.0%)
II	4 (1.4%)
IIA	3 (1.0%)
IIB	3 (1.0%)
IIC	7 (2.4%)
III	19 (6.6%)
IIIA	3 (1.0%)
IIIB	4 (1.4%)
IIIC	58 (20.0%)
IV	56 (19.3%)
Treatment given	
Chemotherapy	244 (84.1%)
No chemotherapy given	46 (15.9%)

**Figure 1:**
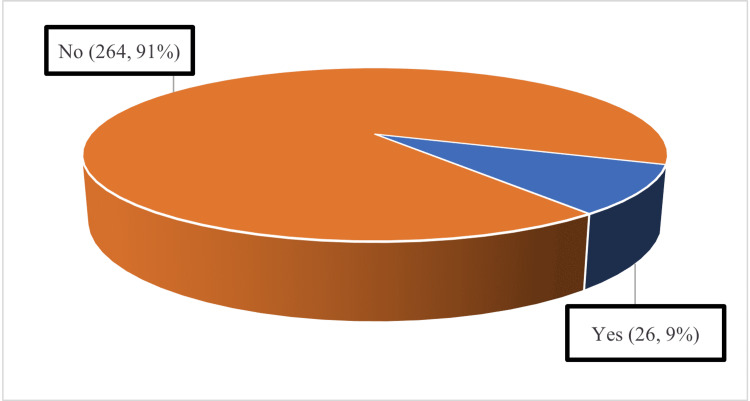
Recurrence of disease after treatment during follow-up

Out of 290 cases, 26 (9.0%) had a recurrence of the tumor while 264 (91.0%) did not have a recurrence of the tumor (Figure 1).

Out of 290 cases, 46 (15.9%) died during follow-up, 129 (44.4%) had disease-free survival while 115 (39.7%) were fine till the end of the study. The mean duration of survival was 3.56 ± 2.33 years. When the survival of patients was compared in the treatment groups, the survival of patients who did not receive chemotherapy was better than in patients who received chemotherapy (Table 2).

**Table 2:** Survival of patients during follow-up

	F (%)
Death	46 (15.9%)
Disease-free survival or Relative survival till the end of the study	244 (84.1%)
Survived up to	
2011	1 (0.3%)
2012	7 (2.4%)
2013	6 (2.1%)
2014	6 (2.1%)
2015	10 (3.4%)
2016	16 (5.5%)
2017	23 (7.9%)
2018	27 (9.3%)
2019	32 (11.0%)
2020	32 (11.0%)
2021	130 (44.8%)
Duration of survival	3.56 ± 2.33

## Discussion

Germ cell cancers of the ovary can be cured and treated successfully. Chemotherapy's long-term effects on long-term survivors remain unknown, although these patients can expect to live normally. Early detection and multi-agent chemotherapy are linked to high cure rates of 85.6 percent (range 81.2-90.0%) in female malignant ovarian germ cell tumors [14-15]. Although male germ cell tumors, which are 20 times more common than malignant ovarian germ cell tumors, share many similarities, women who recur with this malignancy have a worse prognosis [16].

Germ cell tumors are more likely to affect adolescents and women of reproductive age [17]. As in our study, we observed that the mean age of patients was 21.45 ± 9.28 years. Out of 290 cases, the maximum number of patients falls in the age group 20-40 years, which is the reproductive age group.

In our study, we observed that ovarian malignancy was involved in 245 (84.5%) patients while uterine malignancy was noted in 44 (15.2%) cases, and there was only one (0.3%) case of cervical carcinoma. The most common stage at diagnosis of malignancy was IA (96 (33.1%)), followed by IIIC (58 (20.0%)), IV (56 (19.3%)), and IC (26 (9.0%)). Chemotherapy was given in 244 (84.1%) cases. Out of 290 cases, 26 (9.0%) had a tumor recurrence, while 264 (91.0%) did not have a recurrence of the tumor. Out of 290 cases, 46 (15.9%) died during follow-up, 129 (44.4%) had disease-free survival, and 115 (39.7%) were fine till the end of the study. The mean duration of survival was 3.56 ± 2.33 years. When the survival of patients was compared in treatment groups, the survival of patients who did not receive chemotherapy was better than patients who received chemotherapy.

Except for dysgerminoma, for which the incidence of bilaterality is 10-15%, bilateral ovarian germ cell tumors are exceedingly rare [18]. In our study, dysgerminoma was detected in 31.7% of cases with choriocarcinoma in 16.2% cases, immature teratoma in 9.7%, and malignant teratoma in 1.0% cases while yolk sac tumor was detected in 22.4% cases. Several studies have shown that normal reproductive function can be maintained without jeopardizing survival [19-20].

While chemotherapy can damage ovarian function and cause ovarian or premature ovarian failure, most women who receive platinum-based therapy for three or four cycles regain normal ovarian function, and fertility is often preserved in this group [21-23]. In another cohort of 71 patients treated with fertility-sparing surgery and combination chemotherapy, the impact of platinum-based chemotherapy on adult women's ovarian function was documented (including cisplatin and bleomycin). Sixty-two (87%) of these women were able to resume regular menstruation, and 24 of them went on to have 37 offspring [24].

The function of vigorous cytoreduction in advanced illness is unclear, and removing both ovaries does not improve the result. Combining bleomycin, etoposide, and cisplatin is considered the gold standard for adjuvant therapy. Studies of ovarian and reproductive ability after conservative surgery and chemotherapy for malignant ovarian germ-cell tumors have repeatedly shown that these women have an excellent prognosis, with regular menstrual function and fertility rates returning with no increased risk of teratogenicity [25]. Because of the physiological similarities between ovarian and testicular germ cell tumors, Tewari claims that the evolution of systemic treatment for ovarian germ cell cancer has matched advances in the treatment of testicular germ cell cancers [26].

Moreover, all data showed that most women with ovarian germ cell cancers have an excellent survival rate of 93% with effective adjuvant therapy (chemotherapy) and conservative surgery [26-27]. After treatment, they will keep their menstruation and reproductive capacity [27]. According to Joliniere et al., the efficacy of chemotherapy allowed for conservative surgery, such as unilateral salpingo-oophorectomy, while preserving fertility. Chemotherapy was indicated for non-dysgerminoma tumors following surgical staging and debulking. The type of tumor and its histological features influenced the treatment options [28].

There were certain limitations to our study. First, this was a retrospective study with a small sample size. Second, we did not have data on the use of fertility-sparing therapy or on the long-term effects of chemotherapy on ovarian function or secondary malignancies. Third, our study was based on a single institution's experience and may not be generalizable to other centers.

## Conclusions

Overall survival was good during the 10-year follow-up period, with a rate of over 20%. Chemotherapy has improved the outcome of many carcinomas thanks to advances in research, science, and technology. However, we found contradictory results in this study, which could be due to advanced-stage disease at chemotherapy initiation. Further research with larger sample size and other parameters that may affect female survival should be conducted.
